# Clinical Features, Antibiotic Susceptibilities, and Outcomes of Endophthalmitis Caused by Streptococcal Infection: Children vs. Adults

**DOI:** 10.3390/antibiotics12060962

**Published:** 2023-05-25

**Authors:** Yao Yang, Yuenying Wong, Yujie Li, Fang Duan, Xinqi Ma, Hiufong Wong, Rongsha Sun, Jieting Zeng, Manli Liu, Zhaohui Yuan, Xiaofeng Lin

**Affiliations:** State Key Laboratory of Ophthalmology, Zhongshan Ophthalmic Center, Sun Yat-sen University, Guangzhou 510060, China; yangy573@mail.sysu.edu.cn (Y.Y.);

**Keywords:** endophthalmitis, *streptococcus*, antibiotics, visual outcomes, epidemiology

## Abstract

*Streptococcus* spp. are common causative organisms of endophthalmitis. Analysis of the clinical features, antibiotic susceptibilities, and outcomes of streptococcal endophthalmitis in children and adults may guide future management. Sixty-seven patients (67 eyes) with streptococcal endophthalmitis who were admitted to the Zhongshan Ophthalmic Center between January 2013 and December 2022 were retrospectively reviewed. The mean age was 20.7 ± 21.6 years, and 59.7% were children. Streptococcal infection accounted for 13.9% of culture-proven bacterial endophthalmitis cases; the proportion was higher in children than in adults (32.3% vs. 7.6%, *p* < 0.01) and increased from 8.1% in 2013–2017 to 20.1% in 2018–2022 (*p* < 0.01). Eye trauma was the most common etiology in both children and adults (82.5% and 66.7%, respectively). Viridans group streptococci were the most common isolates, followed by *S. pneumoniae.* The susceptibility rates of the streptococci to vancomycin, cefuroxime, and levofloxacin were 100%, 95.5%, and 93.0%, respectively. The overall mean best-corrected visual acuity increased from 2.74 ± 0.19 logMAR initially to 2.32 ± 0.75 logMAR at the last follow-up (*p* < 0.05). In conclusion, streptococcal infections have increased in cases of bacterial endophthalmitis in recent years and are more common in children. The commonly used antibiotics, vancomycin, cefuroxime, and fluoroquinolone, showed higher antibiotic susceptibility. After prompt treatment, visual outcomes improved.

## 1. Introduction

Endophthalmitis is a severe vision-threatening ocular infection resulting from the intraocular colonization of microorganisms and is defined as an ophthalmic emergency owing to its potential for significant visual morbidity and sequelae [1]. Therefore, endophthalmitis treatment is a therapeutic challenge [2].

Based on the mode of entry of the organism, infection is defined as exogenous or endogenous. Depending on the causative event, exogenous endophthalmitis can be post-traumatic, postoperative, or related to keratitis. The incidence of post-traumatic endophthalmitis is estimated to be 0.9–41%, varying geographically [3,4,5]. The incidence of postoperative endophthalmitis after cataract surgery was reported to range from 0.02 to 0.20% [6,7], and trabeculectomy may have a high risk (1.8–5.7%) for delayed or late-onset endophthalmitis [8,9,10]. Endogenous endophthalmitis is a rare complication of systemic blood-stream infections, with an incidence of 0.04–0.4% and accounting for 2–15% of all endophthalmitis cases [11,12,13,14].

*Streptococcus* spp. are the leading cause of late bleb-related endophthalmitis and a common cause of post-traumatic endophthalmitis [15,16]. They are also frequently implicated after post-cataract surgery, vitrectomy, and intravitreal injection [15,17,18,19]. Previous studies found that streptococcal infection was often associated with poor vision prognosis and likely resulted in evisceration/enucleation [6,15,20,21]. Although pars plana vitrectomy (PPV) was known to be effective for endophthalmitis [22,23], the efficacy of early PPV in the treatment of streptococcal endophthalmitis is still controversial [21,24]. The microorganism profile, characteristics, and outcomes of endophthalmitis vary over time and region; however, recent studies with larger samples of streptococcal endophthalmitis are lacking.

In the present study, we reviewed a relatively large sample of 67 cases of streptococcal endophthalmitis with the following aims: (1) to describe the microorganism profile, etiology, and sensitive antibiotics for streptococcal endophthalmitis; (2) to analyze the changing trend in the incidence and microorganism profile and compare the differences between adults and children; and (3) to describe the treatment strategy and analyze visual outcomes. Our findings provide important data that can guide the future management of these sight-threatening conditions.

## 2. Results

During the 10-year study period, 67 cases (67 eyes) of streptococcal infections were identified in 481 patients with culture-proven bacterial endophthalmitis. The mean age of patients was 20.7 ± 21.6 years (ranging from 8 months to 71 years), and the proportion of male to female was nearly 3:1 (50:17). Regarding age distribution, children (≤18 years) accounted for a larger proportion (59.7%, 40/67).

### 2.1. Etiology and Incidence

Based on the etiology, 51 (76.1%) streptococcal infections occurred after eye trauma. Injuries caused by metal tools (52.9%) were the most common, followed by those caused by plants (23.5%). The proportion of post-traumatic endophthalmitis was higher in children (82.5%) than in adults (66.7%) with streptococcal infections. Seven (10.4%) patients were diagnosed with endogenous endophthalmitis; two were children, and five were adults. In four (6.0%) patients with postoperative endophthalmitis, two occurred after cataract surgery, and one occurred after penetrating keratoplasty and trabeculectomy (Table 1).

In this study, streptococcal infections accounted for 13.9% of bacterial endophthalmitis cases, and this proportion was significantly higher in children than in adults (32.3% vs. 7.6%, *p* < 0.01). The proportion of streptococcal infections increased from 8.1% in 2013–2017 to 20.1% in 2018–2022 (*p* < 0.01), and both children (25.0% vs. 39.1%, *p* = 0.09) and adults (2.7% vs. 12.9%, *p* < 0.01) presented the same trend (Table 2).

### 2.2. Subgroup of Organisms and Antibiotic Susceptibility

The causative microorganisms are listed in Table 3. Among the 67 isolates, 39 (58.2%) were viridans group streptococci (VGS), including *Streptococcus mitis* (18, 26.9%), *Streptococcus salivarius* (6, 9%), *Streptococcus sanguinis* (6, 9%), *Streptococcus gordonii* (3, 4.5%), *Streptococcus anginosus* (3, 4.5%), *Streptococcus oralis* (2, 3%), *Streptococcus cristatus* (1, 1.5%), 19 (28.4%) were *Streptococcus pneumoniae*, and 4 (5.9%) were *Streptococcus agalactiae* and *Streptococcus pluranimalium*. VGS was the most common isolate in both children and adults, accounting for 52.5% and 66.7%, respectively; however, the proportion of *S. pneumoniae* was much higher in children than in adults (40.0% vs. 11.1%, *p* = 0.01). We analyzed the changing trends in microbiological profiles between 2013 and 2017 and 2018 and 2022. The proportion of VGS infections increased from 45% to 63.8% (*p* = 0.15), and that of *S. pneumoniae* infections decreased from 35.0% to 25.5% (*p* = 0.43).

Antibiotic susceptibilities of the *Streptococcus* spp. isolates in this study are summarized in Table 4. The susceptibility rate of *Streptococcus* spp. to vancomycin was 100%, whereas that to levofloxacin and ofloxacin was 93.0% and 91.8%, respectively. Among the cephalosporins, *Streptococcus* spp. had the highest sensitivity to cefuroxime (95.5%), with sensitivity rates to ceftriaxone and cefoxitin of 75% and 74.4%, respectively. The susceptibility rate of *Streptococcus* spp. to penicillin was 73.9%, and that to aminoglycoside antibiotics, such as tobramycin and amikacin, was as low as 25.0% and 12.8%, respectively.

### 2.3. Treatment Strategy and Outcomes

All patients were administered topical antibiotics after clinical diagnosis; quinolone antibiotics, such as levofloxacin, gatifloxacin, and moxifloxacin, were used in 92.5% of patients, and vancomycin was used in 10.4% of patients. All patients were administered intravenous antibiotics after diagnosis, among which 81.5% of adults were administered levofloxacin, cefuroxime, or vancomycin; in children, 40.0% were administered cefuroxime or vancomycin. A total of 58 (86.6%) underwent PPV, and the average time from admission to vitrectomy was 1.9 ± 2.4 days. Four patients (6.0%) received only intravitreal antibiotic injections. Five (6.6%) patients developed enucleation due to uncontrollable infection, and none developed panophthalmitis or orbital cellulitis.

In this study, 27 young children were unable to cooperate with visual examination upon admission, and the mean best-corrected visual acuity (BCVA) of the remaining 40 patients was 2.73 ± 0.19 logMAR (range 40/200 to no light perception [NLP]). Forty-eight patients had BCVA records at the last follow-up, eleven patients were lost to follow-up, and eight patients were unable to cooperate with the VA examination at the last follow-up. The mean follow-up time was 18.03 ± 23.33 months (ranging from 5 days to 100 months), the mean BCVA was 2.32 ± 0.76 logMAR (range 20/50 to NLP). Excluding patients who did not cooperate with the visual examination and were lost to follow-up at the initial and last follow-ups, 39 patients had a record of BCVA at the initial and last follow-up, including four patients who developed enucleation. Table 5 shows the changes in the final BCVA from the initial BCVA in these 39 patients. The mean final BCVA was 2.74 ± 0.19 logMAR, which was significantly better than initial BCVA (2.32 ± 0.75 logMAR, *p* < 0.001).

## 3. Discussion

In this study, we analyzed the clinical data of 67 patients with endophthalmitis caused by *Streptococcus* spp. infections in southern China. Our study showed that streptococcal infection accounted for 13.9% of culture-proven bacterial endophthalmitis cases; this proportion was much higher in children than in adults (32.3% vs. 7.6%, *p* < 0.01). Trauma was the most common etiology in both children and adults, and the proportion was much higher in children than in adults (82.5% vs. 66.7%). Our previous study found that *Streptococcus* spp. was the most prevalent isolate in pediatric post-traumatic endophthalmitis, whereas coagulase-negative staphylococci were the dominant pathogens in a predominantly adult population [15]. This may explain the difference in the proportion of streptococcal infections in bacterial endophthalmitis in children and adults. Moreover, the proportion of streptococcal infections increased significantly from 2013–2017 to 2018–2022 (8.1% vs. 20.1%, *p* < 0.01), in both children (25.0% vs. 39.1%) and adults (2.7% vs. 12.9%), suggesting that streptococcal infections have been increasing in recent years and merit clinical attention.

Previous studies have reported that VGS or *S. pneumoniae* is the most common pathogen of streptococcal endophthalmitis, varying in different regions, climates, and endophthalmitis etiologies [20,25]. VGS is commonly found in the oropharynx, respiratory tract, female genital tract, and gastrointestinal tract and is rarely found in normal conjunctival flora [21,26]. In this study, VGS was the most common isolate (58.2%), and its proportion increased from 45% in 2013–2017 to 63.8% in 2018–2022 (*p* = 0.15). *S. pneumoniae* was the second most common isolate, decreasing from 35.0% in 2013–2017 to 25.5% in 2018–2022 (*p* = 0.43). It is worth noting that, in adults, more patients were infected by *S. agalactiae* than by *S. pneumoniae* (14.8% vs. 11.1%). *S. agalactiae* is part of the normal flora of the skin, throat, lower gastrointestinal tract, and female genital tract and usually causes invasive disease, primarily in pregnant and postpartum women, fetuses, and infants [27,28]. However, endophthalmitis caused by *S. agalactiae* is rare, and only a few cases have been reported [28,29]. Our study reported four cases of *S. agalactiae* endophthalmitis, all of which occurred in the last 5 years (2018–2022) in adults, which is of concern.

Previous studies on streptococcal endophthalmitis showed high susceptibility to commonly used antibiotics such as vancomycin, levofloxacin, and moxifloxacin [24,30,31]. Similarly, streptococci in our study showed 100% susceptibility to vancomycin, followed by the second-generation cephalosporins cefuroxime (95.5%) and levofloxacin (93.0%). However, streptococci were less sensitive to third-generation cephalosporins such as ceftriaxone (75.0%) and cefoxitin (74.4%). Chen et al. reported that *S. pneumoniae* had a sensitivity of up to 97% to penicillin; however, our study showed a relatively low susceptibility (73.9%) [30]. Based on these results, it is recommended that vancomycin and levofloxacin be administered topically, levofloxacin or cefuroxime be administered intravenously, and vancomycin be administered via intravitreal injections.

Streptococcal infections have been reported to be associated with poor visual outcomes in previous studies on endophthalmitis. Lu et al. found that streptococcal infections were an independent prognostic factor for poor vision in patients with post-cataract surgery endophthalmitis [6]. Kuriyan et al. found that despite prompt treatment, evisceration/enucleation was performed in 25% of patients, and most patients had poor outcomes [20]. Similarly, Staropoli et al. found that visual prognosis was poor despite early and appropriate antibiotic treatment [32]. Unlike in previous studies, the visual outcomes of the patients in our series improved significantly. The overall mean BCVA increased from 2.74 ± 0.19 logMAR to 2.32 ± 0.75 logMAR at the last follow-up (*p* < 0.05). There were several reasons for the better visual outcomes of our study. First, VGS was the dominant isolate in our study, accounting for 58.2%. VGS species are relatively less virulent than other streptococci because of the lack of virulence factors, such as streptolysins and exotoxins, and may therefore be less damaging to ocular tissues [21]. Previous studies have also found that *S. pneumoniae* or β-hemolytic streptococci were associated with worse visual outcomes compared with VGS [21,30,33,34]. The high proportion of VGS infections may be a factor influencing the improved visual outcomes. Second, at least 92.5% of patients in our study were administered sensitive antibiotics topically after clinical diagnosis, and 81.5% of adults and 40.0% of children were administered sensitive antibiotics intravenously. Four patients (6.0%) were only administered intravitreal vancomycin injection, and 86.6% of patients underwent PPV within 1.9 ± 2.4 days from admission. Early and appropriate antibiotic treatment combined with early PPV may have a positive effect on visual outcomes. Third, our previous studies found that the visual outcomes of pediatric post-traumatic endophthalmitis in preschool children were poor despite prompt treatment [15]. In this study, we excluded patients who did not cooperate with the visual test from the statistical analysis of visual outcomes, which might have had some impact on the final BCVA.

PPV is thought to be an important treatment for endophthalmitis. Early PPV allows the rapid removal of infective and inflammatory loads in the vitreous, thereby reducing further inflammatory damage to the retina. In recent years, several studies recommended early PPV to control infection as soon as possible or to improve vision prognosis [6,23,35]. However, whether early PPV can improve visual outcomes in patients with streptococcal endophthalmitis remains controversial. Kurniawan et al. conducted a retrospective study to investigate the role of early PPV in improving the visual outcomes of streptococcal endophthalmitis during 1997–2012 [21]. In their cohort, most patients had poor visual outcomes; only 30.7% of patients (31/101) underwent PPV and 14.9% of patients (15/101) underwent PPV within 48 h of admission. They found early PPV had no correlation with visual outcome statistically. Yospaiboon et al. retrospectively reviewed 45 patients with streptococcal endophthalmitis during 2012–2016. the proportion of patients who underwent PPV was higher than that in Kurniawan’s study (62.2%, 28/45), whereas only 20% of patients (9/45) had improved vision after treatment [24]. They found that an early vitrectomy performed within 3 days was the only possible predictive factor associated with improved visual outcomes. In the current study, PPV was performed in 86.6% (58/67) of patients, the average time from admission to surgery was as short as 1.9 ± 2.4 days, and the visual outcomes were better than in previous studies. It suggested that timely PPV could benefit visual outcomes of endophthalmitis caused by streptococcal infection.

This study has some limitations. First, the retrospective nature of this study was a limitation. The Zhongshan Ophthalmic Center is a tertiary referral center in southern China, and the patients included in this study were from all over the country, which hindered follow-up. Second, some of the younger children could not cooperate with visual testing, resulting in incomplete data; these data were excluded when the visual outcomes were statistically analyzed, which might have led to bias.

In conclusion, we analyzed the causative microorganisms, clinical characteristics, drug sensitivity, changing trends, and outcomes of streptococcal endophthalmitis in southern China over the past 10 years. Streptococcal infection accounted for 13.9% of bacterial endophthalmitis cases; the proportion was much higher in children than in adults and increased significantly from 2013–2017 to 2018–2022. VGS was the most common isolate, followed by *S. pneumoniae*. *Streptococcus* spp. are highly sensitive to commonly used antibiotics such as vancomycin, levofloxacin, and cefuroxime. With aggressive antibiotics and surgical treatment, visual outcomes improved significantly.

## 4. Materials and Methods

### 4.1. Population

Consecutive medical records of all patients with culture-proven streptococcal endophthalmitis admitted to the Zhongshan Ophthalmic Center, Guangzhou, China, between January 2013 and December 2022 were retrospectively reviewed. This study was performed in compliance with the principles of the Declaration of Helsinki and was approved by the Institutional Ethics Committee of the Zhongshan Ophthalmic Center, Sun Yat-sen University. The requirement for patient consent was waived due to the retrospective nature of the study.

### 4.2. Procedures

Personal information, including age and sex, was recorded. Clinical characteristics were obtained using an electronic medical record system. Medical treatment data, including etiology, treatment regimen, and visual outcomes, were recorded. Clinical diagnosis of endophthalmitis was chiefly based on clinical manifestations, including the presence of corneal edema, hypopyon, anterior chamber cells, and inflammation in the vitreous [36]. The choice of antibiotics initially depends on the ophthalmologist’s empirical judgment of the infection and might subsequently be adjusted according to smear or culture results. Surgical treatments, including the intravitreal injection of antibiotics, PPV, and enucleation, were performed based on the assessment of the ophthalmologist. The average time from admission to vitrectomy and average follow-up time were recorded. BCVA was recorded at the final follow-up visit.

An aqueous/vitreous culture tap was performed on patients clinically diagnosed with endophthalmitis during surgery. Aqueous was aspirated from the limbus using a needle attached to a 1 mL plastic sterile disposable syringe. Vitreous samples were obtained through the pars plana prior to antibiotic injection or vitrectomy using a needle or vitrector. The samples were inoculated in trypticase soy broth (BACT/ALERT^®^ SA and BACT/ALERT^®^ SN, BioMerieux, Inc., Marcy-l’Étoile, France) overnight at 37 °C. Subsequently, the broth was inoculated onto sheep blood agar and potato glucose agar for the growth of bacterial cultures and fungal cultures, respectively [37]. All bacterial isolates were subjected to species identification using a Vitek 2 Compact (BioMérieux, Marcy-l’Étoile, France). Antibiotic susceptibility testing of the isolated bacteria was performed using both the Kirby–Bauer disc diffusion method and the broth dilution method according to the performance standards for antimicrobial susceptibility testing described by the Clinical and Laboratory Standards Institute (CLSI) guideline (https://www.clsi.org/). (1) Using the Kirby–Bauer disc diffusion method, isolated colonies of the organism to be tested were transferred to a stroke-physiological saline solution (SPSS) and adjusted to 0.5 McFarland standard. The standardized bacterial fluid was dipped by a sterile swab and inoculated to the MH agar plate by swabbing the swab three times over the entire agar surface. The plate was rotated approximately 60 degrees each time to ensure an even distribution of the inoculum. The plate was maintained at room temperature for 3 to 5 min to dry the surface of the agar plate. Antimicrobial-impregnated disks which contain antibiotics with different concentration gradients were placed on the surface of the agar with a gap width no less than 24 mm. The plates were incubated at 35 °C for 16 to 18 h. Additionally, the diameter of the area without obvious bacterial growth was measured to determine the sensitivity of bacteria to antibiotics. (2) In the broth dilution method, isolated colonies of the test organism were transferred into sterile inoculation water, mixed gently, and adjusted to 0.5 McFarland standards. The standardized bacterial fluid and the drug-sensitive test culture medium were mixed completely in the ratio of 1:100. Equivalent bacterial fluid mixture (100 μL) was added to the plates with wells containing antibiotics with different concentration gradients and antimicrobial susceptibility reagents. The plates were incubated overnight. Wells without precipitation and color change means no bacterial growth. The lowest antibiotic concentration which successfully inhibited the growth of bacteria (no bacterial growth) was MIC. The following antibiotics were tested in susceptibility analysis: fluoroquinolones (levofloxacin and ofloxacin), cephalosporins (cefoxitin, cefoxitin, and cefuroxime), aminoglycosides (tobramycin and amikacin), penicillin, azithromycin, and chloramphenicol. Bacterial susceptibilities were recorded as “resistant”, “intermediate”, or “sensitive”. For the purpose of this study, “intermediate” and “sensitive” were both considered to be sensitive.

### 4.3. Definitions

The study population was divided into children (≤18 years) and adults (>18 years) based on patient age. All patients were divided into four endophthalmitis groups: posttraumatic, postoperative, endogenous, and keratitis-related. Streptococci were further subdivided into *S. pneumoniae*, VGS, *S. pluranimalium*, *S. agalactiae*, and others (identified only as streptococci). VGS included *streptococcus mitis*, *streptococcus gordonii*, *streptococcus cristatus*, *streptococcus oralis*, *streptococcus anginosus*, *streptococcus salivarius*, *streptococcus sanguinis* [38]. The Snellen best-corrected visual acuity (BCVA) was converted to logMAR for analysis. For BCVA of counting fingers (CF) or worse, the following conversion factors were used: patients with a VA of CF were arbitrarily assigned a logMAR value of 2.6; hand motion, 2.7; light perception, 2.8; and no light perception, 2.9 [39,40]. The VA of patients who lost their eyeballs after enucleation was defined as no light perception.

### 4.4. Statistical Analysis

Statistical analyses were performed using SPSS (version 20.0; IBM, Armonk, NY, USA). The characteristics of the study population and the isolates and susceptibility rates were summarized using means and standard deviations for continuous variables and percentages for categorical variables. Differences in categorical variables were assessed using the chi-squared test. A paired-sample t-test was used to evaluate changes in the final VA from the initial VA. Statistical significance was set at *p* < 0.05.

## Figures and Tables

**Table 1:** Etiology distribution of streptococcal endophthalmitis.

	Post-Traumatic		Endogenous		Postoperative		Keratitis-Related	
	n	%	n	%	n	%	n	%
Age (years)								
≤18	33	82.5	2	5.0	3	7.5	2	5.0
>18	18	66.7	5	18.5	1	3.7	3	11.1
Sex								
male	38	76.0	4	8.0	3	6.0	5	10.0
female	13	76.5	3	17.6	1	5.9	0	0.0
total	51	76.1	7	10.4	4	6.0	5	7.5

**Table 2:** Proportion of streptococcal infections in bacterial endophthalmitis.

	2013–2017		2018–2022		*p* Value
	n/N *	%	n/N *	%	
Age (years)					
≤18	15/60	25.0	25/64	39.1	0.09
>18	5/187	2.7	22/170	12.9	<0.01
total	20/247	8.1	47/234	20.1	<0.01

* n = number of patients with streptococcal endophthalmitis. N = number of culture-proven bacterial endophthalmitis cases.

**Table 3:** Subgroup distribution of *Streptococcus* spp. of endophthalmitis.

	*S. pneumoniae*		VGS		*S. pluranimalium*		*S. agalactiae*		Others	
	n	%	n	%	n	%	n	%	n	%
Age (years)										
≤18	16	40 *	21	52.5	3	7.5	0	0.0	0	0.0
>18	3	11.1	18	66.7	1	3.7	4	14.8	1	3.7
Time periods										
2013–2017	7	35.0	9	45.0	4	20.0	0	0.0	0	0.0
2018–2022	12	25.5	30	63.8	0	0.0	4	8.5	1	2.1
Total	19	28.4	39	58.2	4	6.0	4	6.0	1	1.5

VGS, viridans group streptococci. * The proportion of *S. pneumoniae* in children was higher than that in adults (χ^2^ = 6.621, *p* = 0.01).

**Table 4:** Antibiotic susceptivity of streptococcal endophthalmitis.

	n/N	%
Vancomycin	14/14	100.0%
Levofloxacin	53/57	93.0%
Ofloxacin	45/49	91.8%
Cefuroxime	42/44	95.5%
Ceftriaxone	12/16	75.0%
Cefoxitin	29/39	74.4%
Penicillin	34/46	73.9%
Chloramphenicol	13/15	86.7%
Tobramycin	12/48	25.0%
Amikacin	5/39	12.8%

n: number of antibiotic-sensitive strains. N: number of strains tested for antibiotic susceptibility.

**Table 5:** Comparison of the final BCVA with the initial BCVA.

	Initial BCVA	Final BCVA	*p* Value
Mean BCVA (logMAR)	2.74 ± 0.19	2.32 ± 0.75	<0.001
>20/60	0	1 (2.6%)	
>20/200–20/60	0	10 (25.6%)	
>CF–20/200	1 (2.6%)	0	
CF	2 (5.1%)	7 (17.9%)	
HM	17 (43.6%)	8 (20.5%)	
LP	12 (30.8%)	4 (10.3%)	
NLP *	7 (17.9%)	9 (23.1%)	

BCVA = best-corrected visual acuity; CF = counting fingers; HM = hand motion; LP = light perception; and NLP = no light perception. * Four patients who developed enucleation were included.

## Data Availability

The authors confirm that the data supporting the findings of this study are available within the article. Further inquiries can be directed to the corresponding authors.
